# Supplementary material for: Digitalisation in Craft Enterprises: Perceived Technostress, Readiness for Prevention and Countermeasures—A Qualitative Study
Source: Int J Environ Res Public Health. 2022 Sep 9;19(18):11349. doi: 10.3390/ijerph191811349 (PMC9517128; doi:10.3390/ijerph191811349)
Supplement: Supplementary file 1 [file ijerph-19-11349-s001.zip › Supplementary file S1_Technostress creators.pdf]

Additional file S1

Technostress creators	
**Techno-overload**	High work demands because of using digital technologies due to high work pace, frequent interruptions, multitasking, extended working hours, expectations of response times in digital communication, etc.
**Techno-complexity**	Certain digital technologies are highly complex and challenge the user’s concentration, qualification, feeling of control, which requires additional time in handling complexity
**Techno-insecurity**	Fear of job loss or status deterioration due to the perception that digital technologies or more qualified personnel in terms of handling digital technologies will replace one's own job/position in whole or in part
**Techno-uncertainty**	A constant feeling of uncertainty and ambiguity caused by chronic digital transformation processes or by features of single digital technologies which foster constant change
**Techno-invasion**	Mobile devices enable high flexibility, which can blur boundaries between work and other life domains, causing work-life-conflicts and impair work recovery
**Techno-unreliability**	Stress induced by breakdowns, technical errors, low usability, and poor user experience in certain digital technologies
**Stress in human-machine interaction**	Irritation because of unpredictability of robot or machine behavior or unspecific anxiety towards robots, high complexity of such systems (see techno-complexity)
**Technological workplace surveillance**	New digital technologies can enable a close monitoring of work performance, location and working times, which can provoke distrust and loss of control in the monitored employees

This illustration is according to Ragu-Nathan et. al (2008); Dragano & Lunau (2020)
