# Supplementary material for: Digitalisation in Craft Enterprises: Perceived Technostress, Readiness for Prevention and Countermeasures—A Qualitative Study
Source: Int J Environ Res Public Health. 2022 Sep 9;19(18):11349. doi: 10.3390/ijerph191811349 (PMC9517128; doi:10.3390/ijerph191811349)
Supplement: Supplementary file 1 [file ijerph-19-11349-s001.zip › Supplementary file S2_Interview guide.pdf]

Universitätsklinikum Düsseldorf

Heinrich Heine Universität Düsseldorf

DigiWerk

Interview Guideline

06.11.2019

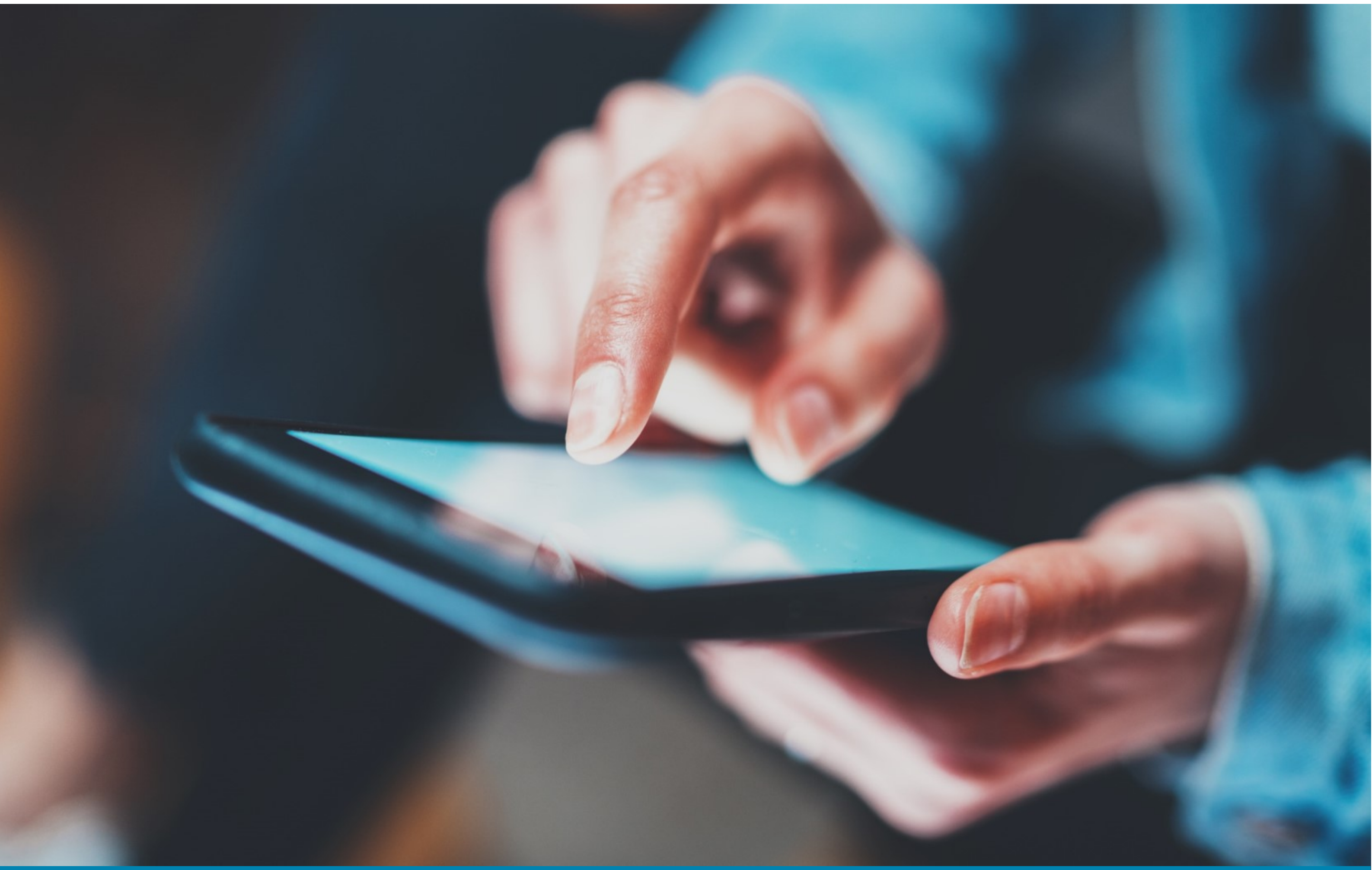

GEFÖRDERT VOM
Bundesministerium für Bildung und Forschung
ESF Europäischer Sozialfonds für Deutschland
EUROPÄISCHE UNION
Zusammen. Zukunft. Gestalten.

- **Eye contact**
- **Let finish**
- **Declaration of consent**
- **Start audio recording**

Before the Interview (Questions to start the conversation)

- Have you had a pleasant day so far?

Personal introduction of the interviewer (name, briefing).

- Would you like to know more about me as a person?
- Discussion of the **participant information**
- **Informed consent form** (written)
- Give participants a short overview of the interview process (topics to be addressed: Digitalisation private and professional, digitalisation and health, work, and health)

Interview (from here the recording of the conversation takes place)

Entry phase:

Professional circumstances and position in the company

Confidence-building question: you work in the ... area of the company (office, field service), is that correct?

- How long have you been working in sanitary, heating and air conditioning sector? (Categories)
- What is a typical working day like for you? (Position in the company, workplace, activities, typical stresses)
  - Please describe your personal workplace (if your own activities have not been dealt with sufficiently before).

Main phase: (Digitalisation)

Personal contact and previous experience with digitalisation

- What do you understand under digitalisation?
- What does digitalisation mean to you? (personal, independent of profession)
  - How important is digitalisation for you personally? (if not already referred to)
- How do you use digital media (smartphone, computer, tablet, etc.) in your everyday life?
- Imagine there is a digitalisation scale from 0 to 10, where 0 means "not digital at all" and 10 means "totally digital" - where would you classify yourself?
  - How do you get to this classification?
  - What would have to happen for you to move up a level?
  - What would have to happen for you to rank yourself at a "10"?

Keep your thoughts on digitalisation in mind. I would now like to discuss your work with you.

Professional use of digital media

- Imagine again the digitalisation scale from 0 to 10, where 0 means "not at all digital" and 10 means "totally digital" - where would you classify the company in which you are employed?
  - How do you get to this classification?
  - What would have to happen for you to move up a level?
  - What would have to happen for you to rank yourself at a "10"?

- At what points in your daily work do you come into contact with digital media or digital work devices?
  - How does this affect your work?
  - Which digital work devices or digital media do you work with so far?
- What concrete steps is your company taking to implement digitalisation in the company? (e.g., training of employees)
  - Can you participate in the process of digitalisation?
    - If "Yes", in what form?
    - If "No", what is preventing you? Or: Would you like to participate in the process?
    - How important is participation in the process of digitalisation in the company to you?
  - What role does it play for you to be able to make your own decisions at work?

- **Eye contact**
- **Let finish**
- **Examples**

Digitalisation and health (resources, stresses, strains)

- Please tell what stresses and strains you personally experience as a result of your work? (Digitalisation, risk of accidents, health risks, stress).
- What connections do you see between your work, digitalisation, and your health?
  - How does digitalisation affect your health and well-being?
- To what extent do you feel burdened by the implementation of digitalisation?
- What positive effects does digitalisation bring to you,
  - in relation to your work?
  - in relation to your health?
- What effects does the use of digital media have on communication with your supervisor and colleagues?
  - To what extent do the changes in communication influence your health?

We have now talked a lot about work and the connection with digitalisation and the impact on your health. Now I would like to talk more about opportunities to stay healthy at work.

Maintaining health at work

- What role does the issue of health play in your company?
- What measures to maintain your health does your company offer you?
  - To what extent do you take advantage of them?
- What do you do every day at work to keep yourself healthy? (also in relation to digitalisation - perhaps revisit stressful factors that have been mentioned).

Wishes for digitalisation

- What are measures related to your health that would support you in the implementation of new work processes (created by digitalisation)? (please describe concrete examples)
  - From whom would you like support in the implementation of digitalisation processes?

- What does healthy work in the age of digitalisation look like in your eyes at your workplace?

You have provided many suggestions and told me a lot about digitalisation, health and work. I would like to thank you for that. But before we end the interview, I have one final question.

Closing phase:

From your point of view, are there still aspects that were not addressed in the interview but are important to you?

- **Eye contact**
- **Examples**
- **Stop audio recording**
- **Farewell**
