# Supplementary material for: Digitalisation in Craft Enterprises: Perceived Technostress, Readiness for Prevention and Countermeasures—A Qualitative Study
Source: Int J Environ Res Public Health. 2022 Sep 9;19(18):11349. doi: 10.3390/ijerph191811349 (PMC9517128; doi:10.3390/ijerph191811349)
Supplement: Supplementary file 1 [file ijerph-19-11349-s001.zip › Supplementary file S3_COREQ.pdf]

Additional file S3

Consolidated criteria for reporting qualitative research (COREQ)

No	Item	Description
**Domain 1: Research team and reflexivity**		
Personal characteristics		
1.	Interviewer	Louisa Scheepers [LS]
2.	Credentials	Louisa Scheepers (M.Sc.), Prof. Dr. med. Peter Angerer [PA], Prof. Dr. Nico Dragano [ND]
3.	Occupation	LS: research associate; PA and ND: university professor
4.	Gender	LS: female; PA, ND: male
5.	Experience and training	LS: educational background in public health, practical experience in occupational health research<br>PA: educational background in in occupational health and public health, practical experience in occupational health research, qualitative and quantitative research and teaching<br>ND: educational background in in epidemiologic and public health, practical experience in occupational health research, qualitative and quantitative research and teaching
Relationship with participants		
6.	Relationship established	No
7.	Participant knowledge of interviewer	Participants knew LS as a researcher and advertisement of the study
8.	Interviewer characteristics	No other characteristics were reported about the interviewer
**Domain 2: Study design**		
Theoretical framework		
9.	Methodological orientation and theory	Qualitative content analysis of Kuckartz (Kuckartz, 2016)
Participant selection		Convenience and snowball sampling
10.	Sampling	Single Interviews
11.	Method of approach	26 participants
12.	Sample size	Not applicable
13.	Non-participation	
Setting		Participants participated in interviews at the location of their workplace
14.	Setting of data collection	No
15.	Presence of non-participants	Between 22 and 62 years (M = 46), 23 males, 3 females
16.	Description of sample	
Data collection		Provided as supplemental material; Interview guide was piloted before study
17.	Interview guide	None
18.	Repeat interviews	Audio recording
19.	Audio/visual recording	None
20.	Field notes	38 – 92 minutes
21.	Duration	Yes
22.	Data saturation	Yes
23.	Transcripts returned	
**Domain 3: Analysis and findings**		
24.	Number of data coders	One (LS)
25.	Description of coding tree	Provided as supplemental material; Examples of the Codebook
26.	Derivation of themes	Deductive coding examples: work and health; digitalisation and work, digitalisation and health
27.	Software	MAXQDA 2020
28.	Participant checking	No
Reporting		
29.	Quotations presented	Yes
30.	Data and findings consistent	Yes
31.	Clarity of major themes	Yes
32.	Clarity of minor themes	Yes

Tong A, Sainsbury P, Craig J. Consolidated criteria for reporting qualitative research (COREQ): a 32-item checklist for interviews and focus groups. International Journal for Quality in Health Care. 2007;19(6):349-357.
