# Supplementary material for: Digitalisation in Craft Enterprises: Perceived Technostress, Readiness for Prevention and Countermeasures—A Qualitative Study
Source: Int J Environ Res Public Health. 2022 Sep 9;19(18):11349. doi: 10.3390/ijerph191811349 (PMC9517128; doi:10.3390/ijerph191811349)
Supplement: Supplementary file 1 [file ijerph-19-11349-s001.zip › Supplementary file S4_Codesystem examples_englisch .pdf]

Additional File S4 – Code List Examples

Code List Examples	Memo	Frequencies
Codesystem		1812
Understanding of digitalisation	Operationalisation:<br>Description of the understanding of digitisation; description of own knowledge or on the topic of digitisation.<br>Coding rules:<br>The entire, detailed description of the understanding of digitisation is coded, both for the private and the professional sphere; descriptions of factual understanding.<br>Anchor example:<br>B12: Yes, well, I personally understand this to mean, roughly speaking, leaving out all this correspondence in paper form and letting everything run via, let's say, clouds or internal systems [...] I understand this to mean, roughly speaking, this.	24
Importance of digitalisation	Name the importance of digitisation for oneself (professionally).<br>Coding rules:<br>Personal attitude, importance of the significance of digitisation for one's own life or person (professional) are coded.<br>Anchor example:<br>B12: For me, well, for me it is already important to work in a resource-saving way, and then accordingly to use less paper, no? Of course, it's an advantage if everything can be done digitally.	27
Fears/ worries about using the technology	Operationalisation:<br>Description of fears in dealing with new digital technologies in private everyday life.<br>Coding rules:<br>All described concerns and fears regarding the use of digital media in private everyday life are coded; resistance to the acquisition or use of new technology in private life is also coded.<br>Anchor example:	5

	B13: But I personally don't think much of it. Yes, you hear a lot about it, don't you? That you can also be hacked at home and that your house is operated by strangers.	
Participation in the digitisation process	This question was only asked of customer service technicians and commercial employees; as managing directors can logically actively participate in the digitalisation process and make decisions.<br>Operationalisation:<br>Describe to what extent or in what way they can participate in the digitisation process.<br>Coding rules:<br>All statements and descriptions of the extent and manner in which participation in the digitisation process is possible are coded; it is also coded if no participation is possible.<br>Anchor example:<br>B12: Well, I don't know myself, but um (...), no, not really (laughs). [...] Well, I mean, if I were asked, I wouldn't say no, but it's not really important for me to participate.	15
Practical training in the technology	Operationalisation:<br>Describing how new technologies are introduced into everyday work.<br>Coding rules:<br>Everything mentioned in connection with learning a technology/software is coded here; training courses; familiarisation periods; contact persons.<br>Anchor example:<br>B13: No, we also have training, so the managing directors, they also take the time, uh, they have already introduced us for several hours, we had also had uh conversations, they also introduced us. [...] And if we had questions, we could also go personally to the boss or junior boss, who rather takes care of it, so we are not completely alone.	24
Introduction of new technologies	Operationalisation:<br>Descriptions of the ways in which new digital media are introduced into the company and everyday working life.<br>Coding rules:<br>Descriptions of the ways in which new digital media are introduced into the company and everyday work are coded - How is the practical introduction carried out? e.g. a test phase ð To be distinguished from the measures and participation digitalisation; here it is about the concrete implementation of the introduction, after the decision has been made; feelings of the employees during the introduction.<br>Anchor example:	6

	B11: now it is the case that we are now in the test phase with it and have bought the devices for the fitters and they will probably start with it from next month, yes. Mhm. [...] (at the same time) Right, exactly, yes. That was always tested, mhm.<br>[...] Yes. Everyone was allowed to take it with them and, uh, everyone tried it out once to see what they thought of it, [...] to get an insight into it.
