# Supplementary material for: Digitalisation in Craft Enterprises: Perceived Technostress, Readiness for Prevention and Countermeasures—A Qualitative Study
Source: Int J Environ Res Public Health. 2022 Sep 9;19(18):11349. doi: 10.3390/ijerph191811349 (PMC9517128; doi:10.3390/ijerph191811349)
Supplement: Supplementary file 1 [file ijerph-19-11349-s001.zip › Supplementary file S5_Codesystem_german.pdf]

Additional File S5 - Liste der Codes

Liste der Codes	Memo	Häufigkeit
Codesystem		1812
Sonstiges		13
Allgemeine Infos zum Berufsstand	Operationalisierung:<br>Allgemeine Informationen zur Branche; zum besseren Verständnis der Branche<br>Codierregeln:<br>Kodiert werden alle allgemein beschreibenden Informationen zum besseren Verständnis der Branche; ohne inhaltlich entscheidenden Bezug zu anderen Themen im Interview, z.B. Themen wie dem Unterschied Monteur und Kundendienstmonteur<br>Ankerbeispiel:<br>B12: Also Kundendienstservice ist im Prinzip äh sage ich jetzt einfach mal, wie ich schon gesagt habe, Service, äh, bedeutet äh Wartung von den Anlagen oder Reparaturen und sowas, und Baustelle ist halt wirklich äh, wie ich eben schon erklärt hatte, die äh alte Anlage rausreißen, neue einbringen oder halt äh Sanitärarbeiten	7
Elternzeit		1
Arbeitsplatz	Oberbegriff: keine Kodierungszuordnung (Memo L = Leer)	0
Fachkräftemangel		1
Arbeitsalltag Rahmenbedingungen	Operationalisierung:<br>Beschreibungen der Rahmenbedingungen am Arbeitsplatz<br>Codierregeln:<br>Kodiert werden allgemeine Beschreibungen zu den Rahmenbedingungen am Arbeitsplatz, die keine typische Tätigkeiten oder ausführenden Arbeit wie z. B. den Wartungsarbeiten entspricht; sondern eher Arbeitszeiten<br>Ankerbeispiel:<br>B10: Ja, ich sage mal, es ist relativ standardmäßig von ähm 7:20 Uhr bis 16:00 Uhr, die normale Dienstzeit, und ähm, ja, man kommt morgens an, bespricht die ähm Mappen, die	18

	Aufträge, die eingegangen sind und eventuell auch das vom vorigen Tag, wenn da was noch nicht fertig ist und ähm dann nimmt man sich die neuen Mappen mit und beginnt sage ich mal sein Werk.	
Rolle/Tätigkeitsbereich im Betrieb	Oberbegriff: keine Codierungszuordnung (Memo L = Leer); es geht aber rein um die zugewiesene Rolle im Betrieb, unabhängig von den Tätigkeiten	0
Geschäftsführer	Operationalisierung:<br>Beschreibung zur Rolle im Betrieb<br>Codierregeln:<br>Kodiert werden Aussagen zur Rolle als Geschäftsführer/Inhaber des Betriebes<br>Ankerbeispiel:<br>noch kein Ankerbeispiel vorhanden<br><br>Interview_26 - ist auch ein Geschäftführer, aber aufgrund von Zeitlichem Engpass habe ich die Frage so explizit im Interview nicht gestellt.	11
Meister, Führungsrolle	Operationalisierung:<br>Beschreibung zur Rolle im Betrieb<br>Codierregeln:<br>Kodiert werden Aussagen zu Führungsaufgaben und Rolle als Meister<br>Ankerbeispiel:<br>B11: Ja, es ist in anderen Bereichen oder wär=wär der Betrieb kleiner, könnte ich wahrscheinlich sagen, ich wäre hier der Chef<br>B11: bin selber Meister und fahre auch manchmal raus	4
Kundendienstmonteur	Operationalisierung:<br>Beschreibung zur Rolle im Betrieb<br>Codierregeln:<br>Kodiert werden Aussagen zur Rolle im Kundendienst/Service/Elektrotechnik (da hier auch bei den Kunden vor Ort an der Anlage gearbeitet wird)<br>Ankerbeispiel:	7

	B13: Ich bin im Kundendienst tätig	
Baustellenmonteur	Operationalisierung:<br>Beschreibung zur Rolle im Betrieb<br>Codierregeln:<br>Kodiert werden Aussagen zur Rolle als Heizungsbauer/Anlagenbauer/Baustellenmonteur<br>Ankerbeispiel:<br>B12: Ich bin hauptsächlich im Bereich Heizungssanierung tätig, also alte Anlagen rausreißen und neue einbringen.	1
kaufm. Angestellte/r	Operationalisierung:<br>Beschreibung zur Rolle im Betrieb<br>Codierregeln:<br>Kodiert werden Aussagen zur Rolle als Bürokraft/kaufmännisch Angestellter (wenn als Haupttätigkeitsfeld benannt); nicht kodiert werden hier Aussagen oder Personen die als Geschäftsführer oder Meister/Führungsrolle tätig sind<br>Ankerbeispiel:	5
typische Tätigkeiten	Oberbegriff: keine Codierungszuordnung (Memo L = Leer); hier geht es ausschließlich um die Tätigkeiten, unabhängig von der Rolle im Betrieb	0
Sanitärbereich		2
Badplanung		5
Renovierungen		1
Arbeiten am Wochenende		3
Kundendienst	Operationalisierung:<br>Beschreibung der anfallenden Tätigkeiten, die ausgeführt werden; Handlungsabläufe<br>Codierregeln:<br>Kodiert werden Beschreibungen zu Tätigkeiten im Rahmen des Kundendienstes, die mit dem Service oder dem Kontakt zum Kunden zusammenhängen; keine Wartungs- oder	8

	Störungsarbeiten<br>Ankerbeispiel:<br>B11: Ja, äh, richtig, also ich habe zwar auch noch ein Auto draußen und Werkzeug, genau, aber überwiegend, ich sage mal 70 Prozent bin ich hier drin, 30 Prozent draußen, ja.	
Reperatur- und Wartungsarbeiten	Operationalisierung:<br>Beschreibung zu Reparatur- und Wartungsarbeiten<br>Codierregeln:<br>Kodiert werden Beschreibungen zu jeglichen Arten von Reparatur- und Wartungsarbeiten; gezielte Beschreibungen zum Ablauf einzelner Wartungsarbeiten werden unter „Ablauf Wartungsarbeiten“ codiert<br>Ankerbeispiel:<br>B13: Äh, häufig sind Wartungen, da wir auch Wartungsverträge haben	12
Hersteller kontaktieren/einbeziehen		1
Ablauf Wartungsarbeiten	Operationalisierung:<br>Beschreibung zu Reparatur- und Wartungsarbeiten<br>Codierregeln:<br>Hier wird alles codiert, was beschrieben wird, wie eine typische Wartung abläuft/welche Tätigkeiten und Arbeiten anfallen oder Zeitaufwand<br>Ankerbeispiel:<br>B13: Heizungsanlage wird gereinigt, äh, dann werden Sicherheitsgruppen geprüft, dann wird der Brennkammer gereinigt und überprüft und äh ja, ob alles äh in Funktion ist, ob alles dicht ist und dann am Ende zum Schluss wird dann noch eine Abgasmessung gef- durchgeführt.	6
Notdienst		9
Elektrotechnik	Operationalisierung:<br>Beschreibung der anfallenden Tätigkeiten, die ausgeführt werden; Handlungsabläufe<br>Codierregeln:	3

		Kodiert werden Beschreibungen zu Tätigkeiten im Zusammenhang mit der Elektrotechnik an Heizungsanlagen<br>Ankerbeispiel:<br>B10: Ähm, ja, prüfen der ähm Endgeräte, also Spannung messen, ähm, ob der Druck vorhanden ist in den Anlagen, ob die Anlage (gibt in Absatz 10 an, dass er für Elektrotechnik zuständig ist)	
	Heizungsbau	Operationalisierung:<br>Beschreibung der anfallenden Tätigkeiten, die ausgeführt werden; Handlungsabläufe<br>Codierregeln:<br>Kodiert werden Beschreibungen zum Ein- und Ausbau von Heizungsanlagen; Neubauten; Abrissarbeiten<br>Ankerbeispiel:<br>B12: […], also alte Anlagen rausreißen und neue einbringen. […] Ich sage mal=ich sage mal ungefähr für eine etwas größere Einfamilienana- Anlage, eine bodenstehende Kesselanlage werden so meistens drei Tage eingeplant. Am ersten Tag wird dann die alte Anlage rausgerissen, ähm, wenn es gut läuft noch der Kamin, also das neue Abgassystem eingezogen, und dann am zweiten Tag äh Einbringen der neuen Kesselanlage, Rohrleitungen anschließen und dann am zweiten Tag meistens noch elektrische Arbeiten und ein paar Restarbeiten. Und Isolierarbeiten und sowas.	6
	Anlagen digitalisieren	Operationalisierung:<br>Beschreibung der anfallenden Tätigkeiten, die ausgeführt werden; Handlungsabläufe<br>Codierregeln:<br>Kodiert werden Beschreibungen wie Heizungsanlagen digitalisiert werden<br>Ankerbeispiel:<br>B11: Und ähm was wir auch , was ich auch mache ist im Grunde mein Steckenpferd, die Anlagen zu digitalisieren, die wir einbauen, das hei- äh sprich, äh, die online schalten und diese halt auch überwachen	1
	E-Mails bearbeiten	Operationalisierung:<br>Beschreibung der anfallenden Tätigkeiten, die ausgeführt werden; Handlungsabläufe<br>Codierregeln:	1

	Kodiert werden Aussagen zum Umgang und der Bearbeitung von E-Mails im beruflichen Setting; nicht jedoch die Aussagen zur ständigen Erreichbarkeit (extra Code – ständige Erreichbarkeit)<br>Ankerbeispiel:<br>B11: Dann schaue ich mir die E-Mails an, was so an Notfälle übers Wochenende, über den ähm , über=über die Nacht reingekommen ist und äh genau, schau dann nochmal, ob wir da reagieren müssen.	
Führungsaufgaben	Operationalisierung:<br>Beschreibung der anfallenden Tätigkeiten, die ausgeführt werden; Handlungsabläufe<br>Codierregeln:<br>Kodiert werden Informationen/Beschreibungen im Umgang mit Mitarbeitern (z. B. Arbeitseinteilung, Gespräche); jedoch keine Aussage die die Art und Weise der Kommunikation zwischen Führungskraft und Mitarbeiter beschreiben (Code – Kommunikation mit Führungskraft)<br>Ankerbeispiel:<br>B11: Ansonsten teile ich morgens dann die Leute ein beziehungsweise erkläre denen w-wenn mal irgendwo komische Sachen sind, wo die einfach nochmal ein Hintergrundwissen haben müssen, äh, dass der Kunde vielleicht schwierig ist oder dass da besonders aufgepasst werden muss, dann bespreche ich das mit den Monteuren und ähm ja, lasse die dann los	23
Akquise der Kunden		1
Baustellenbetreuung		11
Fahrten zum Kunden	Operationalisierung:<br>Beschreibung der anfallenden Tätigkeiten, die ausgeführt werden; Handlungsabläufe<br>Codierregeln:<br>Kodiert werden Aussagen zu Fahrten, die mit dem Kunden verbunden sind (An- und Abfahrt); jedoch keine Angaben zum Thema Zeitdruck/Termindruck<br>Ankerbeispiel:<br>B10: häufig wiederholt ist halt äh die Fahrt zum Kunden, also heißt […] äh immer wieder	2

	hin und zurück	
Vorbereitung auf den Kunden	Operationalisierung:<br>Beschreibung der anfallenden Tätigkeiten, die ausgeführt werden; Handlungsabläufe<br>Codierregeln:<br>Kodiert werden Angaben zur Rüstzeit, Teilebeschaffung für die Baustellen/Kunden aus dem Lager und das Packen der Dienstfahrzeuge, Vorbereitungszeit<br>Ankerbeispiel:<br>B13: Also wir fangen erstmal an hier auf der Firma, dann bereiten wir uns auf die Kunden vor, auf die Termine, ähm, dann packen wir halt all die Dinge ähm mit ins Auto ein, die wir für den Tag brauchen.	2
Büroarbeit	Operationalisierung:<br>Beschreibung der anfallenden Tätigkeiten, die ausgeführt werden; Handlungsabläufe<br>Codierregeln:<br>Kodiert werden Angaben/Beschreibungen zu Zeiträumen, Tätigkeiten (z. B. Angeboteschreiben, Rechnungen, Ersatzteile bestellen (über den Stand-PC; nicht codiert werden hier Aussagen zu Bestellungen über Tablets), Kundengespräche, Buchhaltung – auch bei den Monteuren<br>Ankerbeispiel:<br>B11: Reklamationsbearbeitung über Kundenannahme, Angebote, Rechnungen, ähm, sehe ich eigentlich hier alles und mache alles.	30
Arbeitszettel	Kodiert wird die Beschreibung und Nutzung von Arbeitszetteln<br><br>Ankerbeispiel:<br>B23: So, Arbeitszettel. Also, ich kann hier … also, wie gesagt, dann, dann, wenn wir jetzt irgendwo hinfahren, wird so n Auftragszettel geschrieben, da kommt dann oben Name, Kontaktdaten vom (2) vom Kunden halt hin und dann kommt dann halt oben, hab ich noch ne Zeile, was da (.) los ist. Wenn der das am Telefon sagen kann. Ne, und wenn da halt ne Wartung ist, steht dann da Wartung. (I: mhm) Und dann schreib ich halt mein Bericht, was ich da gemacht habe. (.) (I: mhm) Ne, eventuelles Material wird dann aufgeschrieben. (.) Und dann werden unten dann die Arbeitszeiten erfasst und aufgeschrieben. Und dann lass	5

	ich mir das vom Kunden unterschreiben. (I: mhm) Und hab dann meine … kann dann daraufhin die Rechnung machen.	
Terminierung von Terminen	Operationalisierung:<br>Beschreibung der anfallenden Tätigkeiten, die ausgeführt werden; Handlungsabläufe<br>Codierregeln:<br>Kodiert werden Beschreibungen über den Ablauf der Terminierungen von Terminen (z. B. mit welcher Software, Kommunikation zu Terminen, wer terminiert?)<br>Ankerbeispiel:<br>B10: (gleichzeitig) Genau, also das Büro gibt mir schon Termine vor, ich plane mich teilweise auch schon selber ein, wenn ich weiß so, ja das , da muss ich nochmal hin oder da bin ich nicht fertig geworden oder so, das äh passt dann auch. Und äh , ja, sonst versuche ich das schon relativ zeitig äh abzuwickeln am Ende des Arbeitstages, das man zumindest immer so einen Punkt hat, wo man am nächsten Tag wieder ansetzen kann.	12
Ersatzteilbestellung	Operationalisierung:<br>Beschreibung der anfallenden Tätigkeiten, die ausgeführt werden; Handlungsabläufe<br>Codierregeln:<br>Kodiert werden Beschreibungen über den Ablauf der Ersatzteilbestellung, auch über Tablets oder Handy-Apps; Aussagen im Zusammenhang mit der Digitalisierung und Veränderungen oder Schnittstellenproblematiken werden hier nicht codiert<br>Ankerbeispiel:<br>B10: Äh, bei mir ist es relativ gemittelt, also 50:50 kann man fast sagen, halb Büro halb ähm draußen, weil ich auch für die Ersatzteilbestellungen zuständig dann bin	15
Störungen (Anlagenstörungen)	Operationalisierung:<br>Beschreibung zu Anlagenstörungen<br>Codierregeln:<br>Kodiert werden Beschreibungen zu jeglichen Arten von Anlagenstörungen (elektrisch, digital, mechanisch) und deren Ablauf<br>Ankerbeispiel:<br>B13: Äh, Brennerstörungen meistens. Oder Bauteile, die kaputt gegangen sind, Verschleißteile zum Beispiele. […] Also die Flamme wird ja über äh , überwacht, häufig ist dann die Überwachungselektrode hinne, die nicht mehr in Funktion ist und das führt auch	5

	ziemlich häufig zu Störungen oder der Wasserdruck zum Beispiel.	
Beschäftigungszeitraum	Operationalisierung:<br>Nennung des Zeitraumes der Betriebszugehörigkeit; Nicht in allen Interviews wurde die Frage gestellt, weil dies auch im Kurzfragebogen erfasst wurde<br>Codierregeln:<br>Kodiert wird der Zeitraum der Betriebszugehörigkeit<br>Ankerbeispiel:<br>B10: Ja, das sind jetzt circa anderthalb Jahre, die ich hier bin.	5
Verständnis von Digitalisierung	Operationalisierung:<br>Beschreibung des Verständnisses von Digitalisierung; Beschreibung des eigenen Wissens oder zum Thema Digitalisierung<br>Codierregeln:<br>Es wird die gesamte, ausführliche Beschreibung zum Verständnis von Digitalisierung kodiert, sowohl für den privaten als auch den beruflichen Bereich; Beschreibungen zum Sachverstehen<br>Ankerbeispiel:<br>B12: Ja, also ich persönlich verstehe da drunter jetzt äh im Groben sage ich mal dieses ganze , diesen ganzen Schriftverkehr in Papierform wegzulassen und dass man da alles über irgendwelche sage ich mal Clouds oder interne wie hier äh Systeme äh laufen zu lassen [...] Das verstehe ich jetzt so im Groben da drunter.	24
Analoge Typ	Kodiert werden Aussage dazu, dass jemand lieber analog arbeitet und daran auch noch sehr festhält<br><br>Ankerbeispiel:<br>B23: bin ich ja eher der Zetteltyp.	1
Bedeutung von Digitalisierung	Benennung der Bedeutung von Digitalisierung für die eigene Person (beruflich)<br>Codierregeln:<br>Kodiert werden die persönliche Einstellung, Wichtigkeit zur Bedeutung der Digitalisierung	27

	für das eigene Leben oder eigene Person (beruflich)<br>Ankerbeispiel:<br>B12: Für mich , also für mich ist es schon wichtig, dass man äh ressourcenschonend arbeitet, dann dementsprechend auch wenig Papier äh verwendet, ne? Ist natürlich schon von Vorteil, wenn das dann alles so digital ablaufen kann.	
Bedeutung Digitalisierung privat	Operationalisierung:<br>Benennung der Bedeutung von Digitalisierung für die eigene Person (privat)<br>Codierregeln:<br>Kodiert werden die persönliche Einstellung, Wichtigkeit zur Bedeutung der Digitalisierung für das eigene Leben oder eigene Person (privat)<br>Ankerbeispiel:<br>B12: Ich sage mal im privaten Sinne ist es natürlich relativ schwierig, weil man ja Post auch noch immer in äh , in Papierform äh bekommt, aber ich sage was , immer, was möglich ist, äh, mache ich auch dann per E-Mail, ne? Wo ich mir das dann auf dem Computer zu Hause abspeichern kann.	7
Digitalisierung privat	Oberbegriff: keine Codierungszuordnung (Memo L = Leer)	139
Analoge Tätigkeiten	Wird kodiert, wenn jemand gezielt benennt, dass er etwas bewusst nicht digital macht im privaten	3
Digitalisierungsgrad privat	Oberbegriff: keine Codierungszuordnung (Memo L = Leer)	0
Total digital - 10	Operationalisierung:<br>Vorstellungen von einer total digitalen Person, durch die eigene Person oder abstrahiert<br>Codierregeln:<br>Kodiert werden die Beschreibungen zu den Vorstellungen zum Leben einer total digitalen Person; Hier wird alles kodiert, wie sich jemand vorgestellt wird, der total digital (10) ist<br>Ankerbeispiel:<br>B11: Ja, wenn ich alles automatisiere, also [...] wenn ich ähm Handy sagen kann "So, jetzt Rollladen rauf, Rollladen runter, jetzt ähm den Rasensprenger an, Rasensprenger aus", ne?	19

	Auto fahr vor, Garagentor geht auf, wenn das Auto angeht oder sowas, dann würde ich sagen, ok, dann bin ich irgendwo bei einer 10 gelandet.	
Steigerung der Digitalität	Operationalisierung:<br>Vorstellungen, die zu einer möglichen Veränderung des Digitalisierungsgrades führen könnten – Frage nicht in allen Interviews gestellt, da nicht passend bzw. bereits klar benannt wurde, dass kein Veränderungswunsch besteht<br>Codierregeln:<br>Hier wird alles kodiert, was im Zusammenhang mit einer höheren Einordnung auf der Digitalisierungskala steht; persönliche Vorstellung von zunehmender Digitalisierung im Privatleben; ebenfalls wird kodiert, wenn es keinen Wunsch nach einer privaten Steigerung des Digitalisierungsgrades gibt<br>Ankerbeispiel:<br>B11: Weiß ich nicht. Digitales Auto, äh (lacht) […] Weiß ich nicht, ähm, ja. Äh, irgendwelche digitalen Veränderungen, ähm, im Bereich ähm (...) , ja, ähm, weiß ich aber auch nicht, ob man das unbedingt will in seinem Haus da weiter voran zu kommen oder w- […] was weiß ich. Digitale Trainer, um mit deinen Kindern äh besser zu lernen oder sowas, also das äh nutze ich momentan noch nicht. Vielleicht wäre das so der nächste Stepp.	9
Skalenwert Digitalisierung privat	Operationalisierung:<br>Benennung des Skalenwertes zum Digitalisierungsgrad im privaten Bereich<br>Codierregeln:<br>Kodiert wird nur die Nennung des Skalenwertes<br>Ankerbeispiel:<br>B12: Ja, ich würde mal sagen, ähm, so Mittelfeld oder so oder etwas übers Mittelfeld hinaus, 6 vielleicht.	28
Begründung Skalenwert privat	Operationalisierung:<br>Beschreibung der Begründung für die Einschätzung zum Digitalisierungsgrad im privaten Bereich<br>Codierregeln:<br>Kodiert wird die Begründung für die Einschätzung des Digitalisierungsgrades im privaten Bereich	25

	Ankerbeispiel:<br>B12: Ja wie gesagt, äh, ich denke mal, dass ja heutzutage viele äh also relativ digital unterwegs sind, allein schon mit dem Smartphones.	
Art der Technologie privat	Operationalisierung:<br>Nennung der Technologien die im privaten Alltag genutzt werden<br>Codierregeln:<br>Kodiert werden alle Nennungen und Beschreibungen von genutzten digitalen Medien im privaten Alltag<br>Ankerbeispiel:<br>B10: Ja, überwiegend äh Tablet, Smartphone und Laptop halt.	30
Nutzung der Technologie privat	Operationalisierung:<br>Beschreibung der Art und Weise der Nutzung von Technologien die im privaten Alltag<br>Codierregeln:<br>Kodiert werden alle Beschreibungen zu der Art der Nutzung von digitalen Medien im privaten Alltag<br>Ankerbeispiel:<br>B13: ich benutze schon häufig ähm , ich nutze das Onlinedienst [...] E-Mails zum Beispiel,	20
Ängste/ Sorgen bei Nutzung der Technologie	Operationalisierung:<br>Beschreibung von Ängsten im Umgang mit neuen digitalen Technologien die im privaten Alltag<br>Codierregeln:<br>Kodiert werden alle beschriebenen Sorgen und Ängste in Bezug auf die Nutzung von digitalen Medien im privaten Alltag; auch Widerstände in der Anschaffung oder Nutzung neuer Technologie im privaten werden mitkodiert.<br>Ankerbeispiel:<br>B13: Aber ich halte persönlich nix von. Ja, man hört ja auch viel von, ne? Dass man auch im ähm , dass man gehackt wird sage ich mal zu Hause und dass von irgendwelche fremde Leute das Haus bedient wird.	5
Digitalisierung beruflich	Oberbegriff: keine Codierungszuordnung (Memo L = Leer)	0

Nutzung der Technologie beruflich	Operationalisierung:<br>Beschreibung der Art und Weise der Nutzung von Technologien die im beruflichen Alltag<br>Codierregeln:<br>Kodiert werden alle Beschreibungen zu der Art der Nutzung von digitalen Medien im beruflichen Alltag<br>Ankerbeispiel:<br>B13: im Kalender haben wir alle Termine aufgelistet, die wir am Tag fahren und dann haben wir noch äh die Aufträge online in unserem Tablet stehen und damit ähm, ja, bearbeiten wir auch die Aufträge. […] Also wir sind von dem schriftlichen Teil im Kundendienst bereits weg und ist alles digital mittlerweile.	78
Art der Technologie beruflich	Operationalisierung:<br>Nennung der Technologien die im beruflichen Alltag genutzt werden<br>Codierregeln:<br>Kodiert werden alle Nennungen und Beschreibungen von genutzten digitalen Medien im beruflichen Alltag<br>Ankerbeispiel:<br>B13: Äh, wir haben ein Handy und ein Tablet<br><br>Im Kommentar ist vermerkt, wenn die Monteure auf der Arbeit ihr privates Handy benutzen und kein Diensthandy haben	84
Digitalierungsgrad beruflich	Oberbegriff: keine Codierungszuordnung (Memo L = Leer)	0
Total digitaler Betrieb	Operationalisierung:<br>Vorstellungen von einem total digitalen Betrieb, am Beispiel des eigenen Betriebes oder abstrahiert<br>Codierregeln:<br>Kodiert werden die Beschreibungen zu den Vorstellungen zum Arbeiten in einem total digitalen Betrieb; Hier wird alles kodiert, wie sich ein Betrieb vorgestellt wird, der total digital (10) ist<br>Ankerbeispiel:<br>B11: Ja. Das äh fängt dann morgens an, wenn ich irgendwas eingebe, was aus dem , was ich aus dem Lager brauche, wo dann eine Maschine fängt wie in der Apotheke, wo dann	20

	das äh Rez- äh das=das Produkt da auf so einer Leiter runterfällt und sagt "Hier das , nimm das mit jetzt", […] Das ähm wäre nochmal so eine Geschichte. Äh, ja, jeder nur noch mit Pads rumläuft, wir hier gar keine äh Papiere mehr sehen im Büro oder in der Firma. Das äh , ja, wäre so glaube ich der nächste Schritt oder das vollendete Werk dann. Irgendwann. […] Und alles auch miteinander kommuniziert, das wäre traumhaft.	
Steigerung der Digitalität beruflich	Operationalisierung:<br>Vorstellungen, die zu einer möglichen Veränderung des Digitalisierungsgrades im beruflichen führen könnten – Frage nicht in allen Interviews gestellt, da nicht passend bzw. bereits klar benannt wurde, dass kein Veränderungswunsch besteht<br>Codierregeln:<br>Hier wird alles kodiert, was im Zusammenhang mit einer höheren Einordnung auf der Digitalisierungskala im Beruflichen steht<br>Ankerbeispiel:<br>B10: Ja, da müsste das Ganze halt noch mobil anwendbar werden, dass man das halt von unterwegs auch kann. Hier im Büro bin ich natürlich immer äh direkt verbunden mit der ganzen Sache. Äh, draußen habe ich natürlich aktuell noch meine Papiermappen und muss das dann hier erst wieder einscannen, diesen , wenn man diesen Weg nicht mehr gehen müsste, dann wäre es wahrscheinlich noch digitaler, aber ob es dann auch besser ist, das muss man dann sehen.	18
Skalenwert Digitalisierungsgrad beruflich	Operationalisierung:<br>Benennung des Skalenwertes zum Digitalisierungsgrad im beruflichen Alltag<br>Codierregeln:<br>Kodiert wird nur die Nennung des Skalenwertes in Bezug auf die Einschätzung der Digitalität des Betriebes<br>Ankerbeispiel:<br>B12: Ja, schon ziemlich hoch, 8, 9.	24
Begründung Skalenwert beruflich	Operationalisierung:<br>Beschreibung der Begründung für die Einschätzung zum Digitalisierungsgrad im beruflichen Alltag<br>Codierregeln:	25

	Kodiert wird die Begründung für die Einschätzung des Digitalisierungsgrades im beruflichen Alltag; auch konkrete Beispiele, die auch nicht direkt im Zusammenhang mit dem Skalenwert genannt werden<br>Ankerbeispiel:<br>B13: Wir versuchen noch digitaler zu werden, also unser , zum Beispiel unser Lagerbestand, dass das alles ähm digital äh , also wir machen jetzt auch zum Beispiel jetzt Inventuren, das wird alles digital verarbeitet, dass man schon direkt einen Überblick hat, wo ist der Bestand und äh die , alle mögliche – ich würde auch so zwischen 7 und 8, und wir versuchen weiter zu kommen.	
Arbeit und Gesundheit	Oberbegriff: keine Codierungszuordnung (Memo L = Leer)	0
Definition Gesundheit		1
empfundene Belastungen	Oberbegriff: keine Codierungszuordnung (Memo L = Leer)	0
empfundene körperliche Belastungen	Operationalisierung:<br>Beschreibungen von körperlichen Belastungen, die durch die Arbeit erfahren werden<br>Codierregeln:<br>Kodiert werden alle Aussagen und Beschreibungen zu körperlichen Belastungen, die durch die Arbeit empfunden werden oder auftreten<br>Ankerbeispiel:<br>B11: also wie gesagt, klar, Rücken, arbeitstechnisch sind wir hier , ist ja normal in einem Handwerksbetrieb	19
empfundene psychische Belastungen	Operationalisierung:<br>Beschreibungen von psychischen Belastungen, die durch die Arbeit erfahren werden<br>Codierregeln:<br>Kodiert werden alle Aussagen und Beschreibungen zu psychischen Belastungen, die durch die Arbeit empfunden werden oder auftreten; auch indirekte Aussagen, wo eine psychische Belastungen aus der Art und Weise der beschriebenen Tätigkeit hervorgeht; mit und ohne Bezug zur Digitalisierung	26

	ð Konkrete psychische Belastungen durch die Digitalisierung werden zusätzlich codiert unter „empfundene Belastungen Digitalisierung“<br>Ankerbeispiel:<br>B12: Ich würde sagen, wenn die Baustelle vielleicht mal etwas eng geplant ist oder zu , wenn man zu wenig Zeit bekommt oder wenn unvorge- unvorhergesehene Dinge passieren, dann kommt schonmal Stress auf,	
Ressourcen	Operationalisierung:<br>Beschreibungen von Dingen die Rückhalt bieten oder als eine Art Ressource beschreiben werden<br>Codierregeln:<br>Kodiert werden alle Aussagen und Beschreibungen von Dingen die Rückhalt bieten oder als eine Art Ressource beschreiben werden im Zusammenhang mit der Ausübung der Arbeit; z. B. sozialer Rückhalt<br>Ankerbeispiel:<br>Bisher kein Beispiel	0
Rolle der Gesundheit im Betrieb	Operationalisierung:<br>Beschreibungen der Wahrnehmung und Rolle der Gesundheit im Betrieb<br>Codierregeln:<br>Kodiert werden alle Aussagen und Beschreibungen zur Rolle und Wichtigkeit der Gesundheit im Betrieb; ebenfalls getätigte Begründungen für die Aussagen<br>Ankerbeispiel:<br>B13: Gesundheit ist das Wichtigste hier.	28
Thema Rauchen		1
betriebliche Maßnahmen zur Gesunderhaltung	Oberbegriff: keine Codierungszuordnung (Memo L = Leer)	0
Fuhrpark gut gewartet		2
Betriebsarzt Check-Up/Impfungen		4

Gespräche/Unterweisungen durch Geschäftführer		5
Maschinen ohne Rückschlag/Vibration		1
möglichst wenig Überstunden		3
Ergonomische Büroausstattung		5
Arbeitskleidung		2
feste Mittagspause		4
Schulungsmaßnahmen Gesundheit		6
Impfungen		1
Hilfsmittel für Lasten	Operationalisierung:<br>Beschreibungen zu Hilfsmittel bei der Arbeit im Zusammenhang mit schweren Lasten<br>Codierregeln:<br>Kodiert werden Beschreibungen zu Hilfsmittel bei der Arbeit im Zusammenhang mit schweren Lasten<br>Ankerbeispiel:<br>B13: Wir müssen uns auch nicht körperlich äh wirklich belasten, wir kriegen auch die Hilfsmittel, was uns die Arbeiten auch einfacher machen. [...] Äh, ich sage mal so, statt eine Vier-Meter-Leiter rauszuziehen steht uns auch zu, auch mal eine Hebebühne auszuleihen, bevor man vom , von vier Meter vom Leiter fällt so als Beispiel.	5
Vorbild sein		2
gesunde Ernährung	Operationalisierung:<br>Aussagen und Beschreibungen zum Thema gesunde Ernährung<br>Codierregeln:<br>Kodiert werden Aussagen und Beschreibungen zum Thema gesunde Ernährung, z. B. aber auch gemeinsames Mittagessen, gestelltes Essen vom Betrieb, Getränke<br>Ankerbeispiel:<br>B10: Es gibt äh ab und an auch äh Obst und Gemüse, ist hier frei zugänglich. Es gibt äh ausreichend Wasser und äh auch andere Getränke schonmal, also kann ich mich jetzt hier	6

		nicht beklagen.	
	Maßnahmen für positives Betriebsklima	Operationalisierung:<br>Beschreibungen im Zusammenhang mit der Entwicklung oder dem Erhalt eines guten Betriebsklimas<br>Codierregeln:<br>Kodiert werden Beschreibungen im Zusammenhang mit der Entwicklung oder dem Erhalt eines guten Betriebsklimas<br>Ankerbeispiel:<br>B11: wir haben ja Monatsmeetings, wo im Grunde auch festgestellt wird, ähm, was läuft, was läuft nicht, was können wir verbessern, was haben wir vor in Zukunft, wo soll die Reise hingehen und ähm da haben wir eigentlich schon einen stetigen Austausch, was äh , was diese Sachen , was gute Sachen und schlechte Sachen angeht, ja. (...)	12
	Persönliche Schutzausrüstung	Operationalisierung:<br>Aussagen zur Stellung von PSA und Beschreibungen zur Nutzung der PSA<br>Codierregeln:<br>Kodiert werden Aussagen zur Stellung von PSA und Beschreibungen zur Nutzung der PSA<br>Ankerbeispiel:<br>B13: Oder Schutzbrillen oder Ohrschutz, gibt es ja einige Dinge. [...] Sicherheitsschuhe haben wir natürlich auch an, falls uns mal was auf die Füße fällt	18
	Online Schulungen gesundes Arbeiten	Operationalisierung:<br>Aussagen oder Beschreibungen im Zusammenhang mit Online-Schulungen, die sich auf das Thema Gesundheit oder Arbeitsschutz beziehen<br>Codierregeln:<br>Kodiert werden Aussagen oder Beschreibungen im Zusammenhang mit Online-Schulungen, die sich auf das Thema Gesundheit oder Arbeitsschutz beziehen<br>Ankerbeispiel:<br>B13: Wir machen auch eine Onlineschulung, das hat viel mit äh Gefahrengut, Gesundheit und alles Mögliche zu tun, da werden wir nochmal online äh geschult.	4
	Hautschutz	Operationalisierung:<br>Aussagen zum Thema Hautschutz	1

		Codierregeln:<br>Kodiert werden alle Aussagen oder Nennungen zum Thema Hautschutz und Hautschutzmaßnahmen<br>Ankerbeispiel:<br>B10: Mh, ich sage mal Belastungen ist halt, man=man sollte sich erstmal dann Handschuhe anziehen, ne? Ähm, mit verschiedenen Stoffen, die halt , wo die Haut auch mal schonmal drauf reagieren könnte	
	Stressprävention	Operationalisierung:<br>Beschreibungen zu Maßnahmen zur Stressprävention<br>Codierregeln:<br>Kodiert werden alle Nennungen und Beschreibungen im Zusammenhang mit dem Thema Stressprävention; Schulungen; interne Betriebsmaßnahmen<br>Ankerbeispiel:<br>B10: ähm, und dann , ja so eine=so eine IKK-Beratung äh über das Thema äh Stress am Arbeitsplatz und Gesundheit und äh Gesunderhaltung	3
	Rückenübungen/Kurse/Bewegung	Operationalisierung:<br>Beschreibungen zu Bewegungskursen und Rückenübungen<br>Codierregeln:<br>Kodiert werden Beschreibungen zu Bewegungskursen und Rückenübungen; durch Externe Berater aber auch interne Schulungen oder Besprechungen<br>Ankerbeispiel:<br>B11: Also wir haben auch gerade vor Kurzem ähm einen Trainer da gehabt, so einen=so einen äh , von der IKK war der glaube ich, der im Grunde sich um die Fitness, um die Probleme äh gekümmert hat und so sagen kann, um uns halt auch zu helfen, wie können wir einige Sachen verbessern mit ähm, ja, so ein bisschen äh auch Probeübungen, die man dann auch zu Hause vor der Arbeit, nach der Arbeit machen kann, um halt auch die , damit die Fitness und keine großen Rückenbeschwerden da sind, ne? Damit man halt das so ein bisschen besser angehen kann, ne? Ähm, das äh wird sich schon gut drum gekümmert.	18
Krankenkassen gehen auf Betriebe zu			1
Schulungsinhalte nicht umsetzbar im Alltag			1

Veränderungswünche BGF	Operationalisierung:<br>Aussagen und Beschreibungen zu Veränderungswünschen an BGF Maßnahmen oder anderen betrieblichen Maßnahmen zur Gesundheit<br>Codierregeln:<br>Kodiert werden alle Aussagen und Beschreibungen zu Veränderungswünschen an BGF Maßnahmen oder anderen betrieblichen Maßnahmen zur Gesundheit<br>Ankerbeispiel:<br>B11: Ja, ich meine, Reize kann man immer schaffen, ne? Ob es jetzt äh , ich weiß das auch nicht, wie das umsetzbar ist, aber klar, man kann ja immer so ein bisschen äh , jeder hat ja natürlich so einen anderen Wunsch, der eine geht vielleicht gerne ins Fitnessstudio, vielleicht kann man den da unterstützen, der andere geht gerne schwimmen, was ja alles für den Körper ist und was alles gut tut, ähm, ja, äh da gibt es glaube ich noch Potenzial, also klar.	2
Inanspruchnahme der betrieblichen Gesundheitsmaßnahmen	Operationalisierung:<br>Aussagen und Beschreibungen im Zusammenhang mit der Inanspruchnahme von betrieblichen Gesundheitsmaßnahmen<br>Codierregeln:<br>Kodiert werden alle Aussagen und Beschreibungen im Zusammenhang mit der Inanspruchnahme von betrieblichen Gesundheitsmaßnahmen; egal ob die Wichtigkeit der Teilnahme beschrieben wird oder das bisher nicht an BGF Maßnahmen teilgenommen wurde; ebenfalls werden die Gründe mitkodiert<br>Ankerbeispiel:<br>B13: [Vertreter der Krankenkasse] aber der hat das natürlich uns=uns allen nochmal nahegelegt haben, […] dass das sehr wichtig ist. [Teilnahme an Online-Schulungen]	1
keine BGF Maßnahmen	Operationalisierung:<br>Nennungen und Beschreibungen, dass es keine Maßnahmen zur betrieblichen Gesundheitsförderung gibt<br>Codierregeln:<br>Kodiert werden Nennungen und Beschreibungen zur nicht Existenz von BGF Maßnahmen; ebenfalls auch die Wertung dessen<br>Ankerbeispiel:	6

	B11: Ja, das ist auf jeden Fall verbesserungswürdig, also da habe ich äh so nichts hier.[...] Ja. Da gibt es noch Potenzial. (lacht)	
persönliche Maßnahmen zur Gesunderhaltung	Operationalisierung:<br>Beschreibungen von persönlichen Maßnahmen zur Erhaltung der eigenen Gesundheit während der Arbeit<br>Codierregeln:<br>Kodiert werden Beschreibungen von persönlichen Maßnahmen zur Erhaltung der eigenen Gesundheit während der Arbeit; ebenfalls wird kodiert, wenn keine persönlichen Maßnahmen zur Gesunderhaltung ergriffen werden und warum<br>Ankerbeispiel:<br>B10: Ich trinke ausreichend Wasser. […] Das ist äh wichtig.	43
Digitalisierung und Arbeit	Oberbegriff: keine Codierungszuordnung (Memo L = Leer)	0
Sorgen und Ängste Digi		9
Teilhabe am Digitalisierungsprozess	Diese Frage wurde nur den Kundendienstmonteuren und kaufm. Angestellten gestellt; da Geschäftsführer logischer Weise am Digitalisierungsprozess aktiv mitwirken und Entscheidungen treffen können<br>Operationalisierung:<br>Beschreibung in welchem Umfang oder Art und Weise am Digitalisierungsprozess mitgewirkt werden kann<br>Codierregeln:<br>Kodiert werden alle Aussagen und Beschreibungen zum Umfang sowie der Art und Weise, wie am Digitalisierungsprozess mitgewirkt werden; kodiert wird auch, wenn keine Teilhabe möglich ist<br>Ankerbeispiel:<br>B12: Also ich se- selber jetzt nicht, aber ähm (...) , nee, eigentlich nicht (lacht). […] Also ich meine, wenn ich jetzt gefragt werden würde, würde ich nicht nein sagen, aber ist jetzt , […] ist mir jetzt nicht unbedingt wichtig da mitzuwirken.	15

Rolle eigene Entscheidungen treffen	Diese Frage wurde nur den Kundendienstmonteuren und kaufm. Angestellten gestellt; beim Geschäftsführer ist klar, dass er eigene Entscheidungen treffen muss<br>Operationalisierung:<br>Aussagen zur Wichtigkeit eigene Entscheidungen während des Arbeitsprozesses treffen zu können<br>Codierregeln:<br>Kodiert werden alle Aussagen zur Wichtigkeit eigene Entscheidungen während des Arbeitsprozesses treffen zu können; ebenfalls konkrete Beispiele<br>Ankerbeispiel:<br>B12: Ich sage mal, spielt bei uns schon eine ganz große Rolle, weil wir ja auf der Baustelle oft eigene Entscheidungen fällen müssen, sage ich jetzt mal in Hinsicht, ob jetzt äh die Anlage so hinstellen oder so hinstellen oder so aufhängen, die L- Rohrleitungen da hinlegen oder da hinlegen, das ist schon , da müssen wir schon sehr selbstständig arbeiten können.	10
Maßnahmen zur Digitalisierungsumsetzung	evtl. recodieren, wenn es mehr Transkripte/Nennungen gibt<br><br>Operationalisierung:<br>Aussagen und Beschreibungen zu den Maßnahmen, die der Betrieb zur Umsetzung der Digitalisierung unternimmt<br>Codierregeln:<br>Kodiert werden alle Aussagen und Beschreibungen dazu, was der Betrieb macht, um die Digitalisierung voran zu treiben, umzusetzen; kodiert werden angedachte Themen und bereits in Angriff genommene Maßnahmen<br>ð Nicht kodiert werden Aussagen und Beschreibungen zur Einführung der Technik, die z. B. mit der Wissensvermittlung im Umgang mit der neuen Technik zusammenhängen oder die Veränderung von Arbeitsabläufen<br>Ankerbeispiel:<br>B10: Ja, das=das Anschaffen der=der Tablets halt zum Beispiel für die Monteure, das wird ein bisschen vorangetrieben, dass die Umstellung erfolgt, was natürlich auch nicht ganz einfach ist und auch nicht jeder will immer mit einem Tablet dann , der Kunde muss auch da irgendwie drauf unterschreiben.<br><br>B12: aber äh wie gesagt, durch die Tablets äh ist natürlich äh einiges passiert vor	26

		geraumer Zeit, ähm, ja dann, was , kann man das auch? Ja, kann man denke ich auch unter Digitalisierung jetzt äh sagen, haben die äh vor Kurzem Solaranlagen hier aufs Dach äh installieren lassen, wo dann quasi ins Netz eingespeist wird, wo dann das Gebäude von versorgt wird. Wo dann der Juniorchef und der Seniorchef die zwei Elektroautos drüber laden.	
	Ideenentwicklung		10
	praktische Einarbeitung in die Technik	Operationalisierung:<br>Beschreibung der Art und Weise, wie neue Technologien in den Arbeitsalltag eingeführt werden<br>Codierregeln:<br>Hier wird alles kodiert, was im Zusammenhang mit dem Erlernen einer Technologie/Software genannt wird; Schulungen; Einarbeitungszeiträume; Ansprechpartner<br>Ankerbeispiel:<br>B13: Nee, wir haben da auch Schulungen , also die Geschäftsführer, die nehmen sich auch die Zeit, äh, die haben uns schon mehrere Stun- wir hatten auch äh Gespräche gehabt, da haben Sie uns auch eingeführt gehabt. […] Und wenn wir Fragen hatten, dann konnte man auch persönlich zu dem Chef gehen beziehungsweise Juniorchef, der sich da eher drum kümmert, also wir stehen dann nicht ganz alleine.	24
	Einführung neuer Technologien	Operationalisierung:<br>Beschreibungen auf welche Art und Weise neue digitale Medien in den Betrieb und Arbeitsalltag eingeführt werden<br>Codierregeln:<br>Kodiert werden Beschreibungen auf welche Art und Weise neue digitale Medien in den Betrieb und Arbeitsalltag eingeführt werden – Wie läuft die praktische Einführung ab? , z. B. eine Testphase<br>ð Abzugrenzen von den Maßnahmen und Teilhabe Digitalisierung; hier geht es um die konkrete Umsetzung der Einführung, nach getroffener Entscheidung; Empfindungen der Mitarbeiter bei der Einführung<br>Ankerbeispiel:<br>B11: jetzt ist es so der Fall, dass wir jetzt in die Testphase dann , in der Testphase damit	6

	sind und die Geräte jetzt für die Monteure gekauft haben und äh die wohl dann da jetzt ab nächsten Monat circa mit anfangen werden, ja. Mhm. […] (gleichzeitig) Richtig, genau, ja. Das wurde immer getestet, mhm.<br>[…] Ja. Durfte dann jeder mal mitnehmen und äh jeder hat es einmal ausprobiert, was er davon hält, […] Um einen Einblick davon zu bekommen.	
Widerstand gegen Neues	Operationalisierung:<br>Beschreibungen zu Widerständen im Umgang oder Einführung neuer Technik<br>Codierregeln:<br>Kodiert werden Aussagen und Beschreibungen zu Widerständen im Umgang oder Einführung neuer Technik; Gründe für den Widerstand<br>Ankerbeispiel:<br>B10: Ja, das=das Anschaffen der=der Tablets halt zum Beispiel für die Monteure, das wird ein bisschen vorangetrieben, dass die Umstellung erfolgt, was natürlich auch nicht ganz einfach ist und auch nicht jeder will immer mit einem Tablet	4
Neue Arbeitsabläufe	Ist mir nicht wirklich klar, wie ich das gut codieren soll....ergibt sich vielleicht mit weiteren Interviews	2
Wissensvermittlung an den Kunden	Operationalisierung:<br>Beschreibung zur Wissensvermittlung an den Kunden im Umgang mit neuer Heiztechnologie<br>Codierregeln:<br>Kodiert werden Beschreibung zur Wissensvermittlung an den Kunden im Umgang mit neuer Heiztechnologie; Heizung über eine APP steuern; Wer ist dafür zuständig?; Verständlichkeit der APP<br>Ankerbeispiel:<br>B12: Ich sage mal das ist natürlich auch stark vom Alter abhängig, ne? Wenn man jetzt äh ältere Personen hat, ist das schonmal schwieriger, da , die haben da schon Schwierigkeiten das Smartphone zu händeln, ja, und dann auch noch die äh Anlage darüber zu steuern ist oft etwas schwierig, aber ich sage mal bei jüngeren Leuten oder mittleren Alters funktioniert das eigentlich ziemlich gut, weil ich sage mal, das ist eigentlich auch äh , ja, sage ich mal ziemlich selbsterklärend.	11

positive Auswirkungen Digi auf Arbeit	Oberbegriff: keine Codierungszuordnung (Memo L = Leer)	143
Badplanung Kundenpräsentarion		1
Arbeitszeiterfassung pro		3
Werkstoffe werden leichter		2
bessere Bedienbarkeit		1
Online Banking		1
vereinfachte Speicherung Daten	Kodiert werden Informationen zu APPs und deren Speicherungsmöglichkeiten sowie von anderen Softwareanwendungen; wenn diese als positiv beschrieben werden<br><br>Ankerbeispiel:	3
transparentere Daten		1
Weniger Konflikte mit Kunden		4
professionellere Außendarstellung		5
wirtschaftlicher		4
weniger Kosten		3
Technik günstiger geworden		1
gringere Fehlerquote		5
Vereinfachte Organisation/Planungsmöglichkeiten		5
Fernwartung pro & kontra		20
Reaktion vor Störung möglich		2
Vereinfachte Arbeitsabläufe		14

Entspannteres Arbeiten		5
Platzsparender		2
Vereinfachter Informationszugang		24
Zeitersparnis		18
Arbeitsersparnis		17
Freude an neuen Technologien		2
negative Auswirkungen Digi auf Arbeit	Oberbegriff: keine Codierungszuordnung (Memo L = Leer)	0
weniger Bewegung		1
Preisdummping		1
Online Bewertungen		1
Home-Office		3
Digitalisierung am Handwerk vorbei geplant		2
Komplexität der Technik	Hier wird alles kodiert, was im Zusammenhang mit Komplexität und Nutzung von Technologien benannt wird	36
aufwendigere Fehlersuche		5
Technik ist empfindlicher		1
Widerstände Mitarbeiter		13
Umstellung ältere Mitarbeiter		6
Jüngere Mitarbeiter lernen schneller		2
Zeitaufwand Einarbeitung		9

Arbeitszeiterfassung		7
Schnelle Neuerungen		21
Am Zahn der Zeit bleiben		11
Kunden fordern digitalität		5
Schnittstellenproblematik		10
Support im Umgang mit der Technik	Hier werden alle Angaben kodiert über den Zugang zu Support.	18
Fehlende Transparenz zu Daten		1
Angst vor Jobverlust		1
erhöhter Zeitdruck/Schnelllebigkeit		20
Grenzüberschreitende Kunden	Hier wird kodiert, wenn Kunden beispielsweise zu "Unzeiten" anrufen/vorbei kommen	10
Oberflächliche Arbeit	Dadurch entstehen dann z. B. auch vermehrt Fehler	1
Nicht abschalten können		1
Verlust Menschlichkeit		20
Mehraufwand		7
Mehr Büroarbeit		4
Verantwortungen verschieben		5
Techniksicherheit/Hackerangriffe		7
Informationsüberflutung		8
Multitasking		7
ständige Erreichbarkeit		36

Überwachung		19
Überwachung durch den Kunden	Damit sind auch die negativen Auswirkungen der Fernwartung gemeint; Fernwartung noch einmal recodieren!!	6
Kunden informierter		2
Schlechter Empfang		9
Kommunikation		9
dezentrale Führung		1
Veränderung Kommunikation Kunde		27
keine Veränderung Kommunikation Kunden		1
Veränderungen der Kommunikation Kollegen/Vorgesetzte		31
keine Veränderung der Kommunikation Kollegen		3
Digitalisierung und Gesundheit		2
wahrgenomme Zusammenhänge zwischen Digitalisierung-Gesundheit		39
Trennung privat und berufliches	Operationalisierung:<br>Beschreibung wie digitale Medien genutzt werden, in welchem Umfang – im Zusammenhang mit den Grenzen zwischen privatem und beruflichen<br>Codierregeln:<br>Kodiert werden die persönliche Einstellung, Haltung im Umgang zur Nutzung von digitalen Medien, in Bezug auf die Grenze zwischen privat und beruflich<br>Ankerbeispiel:<br>B10: Wir haben äh Firmen äh Telefone und manche benutzen nur das, ich habe aber auch noch ein privates, weil ich das äh eigentlich auch strikt trenne. […] Außer im Notdient halt, da erreicht man mich natürlich dann auch über das Firmenhandy unter der Woche, aber normalerweise lege ich das ab 16 Uhr weg.	30

	Gedanke: Vielleicht ergibt sich ein Zusammenhang mir der ständigen Erreichbarkeit, wenn Mitarbeiter die Diensthandys nach der Arbeit mit nach Hause nehmen.	
sich abgrenzen können		18
gesunderhaltende Arbeitsbedingungen Digitalisierung		29
Keine Idee zum gesunderhaltenden Arbeitsplatz		4
empfundene Belastungen Digitalisierung	Aktuell auch erfassen, wenn konkret benannt wird, dass keine Belastungen empfunden werden - dann lässt sich das später vielleicht differenzierter darstellen.	9
Überforderung mit der Umsetzung der Digitalisierung		1
keine Belastung		1
Gedanklich nicht abschalten können		9
weniger Bewegung		1
Leistungssteigerung		6
keine Leistungssteigerung		1
Arbeitsverdichtung		18
Stressempfinden		34
Auswirkungen auf Wohlbefinden		21
positive Auswirkungen Digitalisierung		5
Unterstützung Digitalisierungsprozess		0
Veränderungswünsche Digitalisierung		29
Keine Veränderungswünsche Digitalisierungsprozess		4
Unterstützung Gesundheit im Betrieb		0

Veränderungswünsche Gesundheit	18
Keine Veränderungswünsche Gesundheit	4
